# Metformin Induces MeCP2 in the Hippocampus of Male Mice with Sex-Specific and Brain-Region-Dependent Molecular Impact

**DOI:** 10.3390/biom14040505

**Published:** 2024-04-21

**Authors:** Khatereh Saei Arezoumand, Chris-Tiann Roberts, Mojgan Rastegar

**Affiliations:** Department of Biochemistry and Medical Genetics, Rady Faculty of Health Sciences, Max Rady College of Medicine, University of Manitoba, Winnipeg, MB R3E 0J9, Canada

**Keywords:** Rett Syndrome, RTT, MeCP2, DNA methylation, adult brain, metformin, hippocampus

## Abstract

Rett Syndrome (RTT) is a progressive X-linked neurodevelopmental disorder with no cure. RTT patients show disease-associated symptoms within 18 months of age that include developmental regression, progressive loss of useful hand movements, and breathing difficulties, along with neurological impairments, seizures, tremor, and mental disability. Rett Syndrome is also associated with metabolic abnormalities, and the anti-diabetic drug metformin is suggested to be a potential drug of choice with low or no side-effects. Previously, we showed that *in vitro* exposure of metformin in a human brain cell line induces *MECP2E1* transcripts, the dominant isoform of the *MECP2* gene in the brain, mutations in which causes RTT. Here, we report the molecular impact of metformin in mice. Protein analysis of specific brain regions in the male and female mice by immunoblotting indicated that metformin induces MeCP2 in the hippocampus, in a sex-dependent manner. Additional experiments confirm that the regulatory role of metformin on the MeCP2 target “BDNF” is brain region-dependent and sex-specific. Measurement of the ribosomal protein S6 (in both phosphorylated and unphosphorylated forms) confirms the sex-dependent role of metformin in the liver. Our results can help foster a better understanding of the molecular impact of metformin in different brain regions of male and female adult mice, while providing some insight towards its potential in therapeutic strategies for the treatment of Rett Syndrome.

## 1. Introduction

Rett Syndrome (RTT) is a postnatal and rare neurodevelopmental disorder that affects 1 in 10,000 to 1 in 15,000 of live-born females. In over 95% of patients, RTT results from *de novo* mutations in the Methyl CpG Binding Protein 2 (*MECP2*) gene located on the X chromosome [1]. The encoded protein MeCP2 is highly expressed in the brain, where it is remarkably abundant in neurons. MeCP2 is an epigenetic factor that binds to methylated DNA in the form of both 5-methylcytosine (5-mC) and 5-hydroxymethylcytosine (5-hmC) [2]. Through binding to methylated DNA, MeCP2 controls gene transcription, with additional regulatory roles in RNA splicing, chromatin looping, chromatin compaction, cell signaling pathways, and protein translation [3,4,5,6,7]. Previous studies from our team have shown that the MeCP2 homeostatic regulatory pathway (*MECP2-BDNF-miR132*) is impaired in the post-mortem tissues of RTT patients [8]. Importantly, inducing MeCP2 and/or BDNF expression in the brain has been introduced in the context of potential therapeutic strategies for RTT [2,9].

Recently, RTT has been recognized as having metabolic components, including the impairment of glucose metabolism [10]. Accordingly, the anti-diabetic drug metformin has gained attention in studies related to Rett Syndrome. In a female mouse model of Rett Syndrome, administration of metformin for 10 days rescued the impaired mitochondrial activity in the brain, albeit with no rescue of motor-control abnormalities or behavioral improvement [11]. Our *in vitro* studies in a human brain cell line have also highlighted that metformin can induce *MECP2E1* (the main isoform in the brain/brain cells) and *BDNF* transcripts [12]. However, the molecular impact of metformin on MeCP2-BDNF proteins and some other RTT-related proteins has not been fully explored.

Here, we report the molecular impact of metformin treatment in male and female mice in different brain regions that are associated with RTT phenotypes. We first show that the intraperitoneal (IP) injection of metformin at 200 mg/kg leads to significant and sex-dependent change in the level of phosphorylated ribosomal protein S6 (RSP6) in the liver, the primary target organ for metformin. Next, we show that metformin is capable of significantly inducing MeCP2 in the hippocampus of male mice, with a similar trend in the cerebellum of both male and female mice; however, these changes do not reach statistically significant levels. Further investigation of the BDNF and the ribosomal protein S6, in both phosphorylated and non-phosphorylated form, confirms that the molecular impact of metformin is region-dependent in the brains of both male and female mice. Metformin has been recently highlighted in the context of other neurodevelopmental disorders that are associated with mental disability, such as Fragile X syndrome and autism [13,14,15]; thus, our results might also be of interest in the context of these life-long disorders.

## 2. Materials and Methods

### 2.1. Ethical Statement

The experimental processes that are reported in this study have been reviewed, evaluated, and approved by “the University of Manitoba Animal Research Ethics Board”. All procedures were completed under our approved animal protocol 18-053.

### 2.2. In Vivo Treatments in Mice and Experimental Design

Mice were maintained with ambient room temperatures of 19–22 °C, with access to food and drinking water, managed by the University of Manitoba Animal Facility Staff. The male and female C57BL/6 mice were purchased from the University of Manitoba Animal Facility. Mice received daily intraperitoneal injections of the vehicle Phosphate Buffer Saline (PBS) or metformin (EMD Millipore Crop, St. Louis, MO, USA, 317240) for 3 weeks at 200 mg/kg concentration. It has been reported that with a dose of 200 mg/kg body weight, metformin passes the blood-brain barrier (BBB) [16].

**Figure 1:**
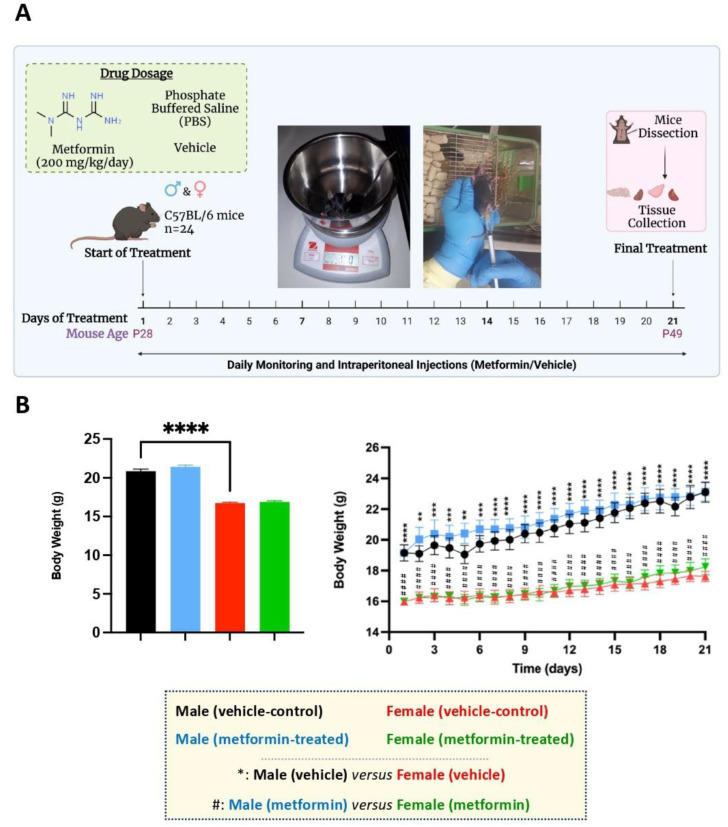
Experimental design and the effect of metformin on the murine average body weight. (**A**) Male and female C57BL/6 mice were treated daily with metformin (200 mg/kg) or vehicle (Phosphate Buffered Saline) by IP injection, starting at P28 for 21 days. At P49, mice were dissected, and tissues were collected. (**B**) As shown in the column graph on the left, sex-difference analysis indicated a significantly higher average body weight of male mice (vehicle) compared to females (vehicle). These analyses were completed by unpaired *t*-test. As shown in the linear graph on the right, significant sex differences were detected between the average body weights of male and female mice at the basal level (vehicle) as well as male and female mice treated with metformin on a daily basis. These analyses were completed by two-way ANOVA followed by Tukey’s multiple comparisons tests. The levels of significance are shown by ** *p* < 0.01, *** *p* < 0.001, **** *p* < 0.0001, and ^####^ *p* < 0.0001, and data are reported as mean ± standard error of the mean (SEM) with N = 6.

For experimental design, please refer to Figure 1A. A total number of 24 mice (12 male and 12 female mice) were randomly categorized into two groups of vehicle control (PBS) and metformin treatment. PBS was used as the solvent for dissolving metformin under sterile conditions. Six male mice and six female mice were treated with metformin (200 mg/kg) through daily IP injections. For controls, the same number of male and female mice were injected with vehicle. IP injections were completed daily, with alternating right and left sides. At the start of IP injections, mice were at postnatal day (P) 28, and were subjected to 21 days of treatment. Mice from all experimental groups were euthanized at P49 and the liver and 4 brain regions (frontal cortex, cerebellum, thalamus, and hippocampus) were dissected as previously reported [17]. These selected brain regions have been reported to contribute to RTT symptoms and/or mechanism of disease, including the frontal cortex [18], cerebellum [19], thalamus [20], and hippocampus [21].

### 2.3. Protein Extraction, Western Blot Experiments, and Statistical Analysis

Total protein extraction and quantification of the murine brain and liver tissues were completed as previously described [22]. Protein extracts (10–30 µg) were subjected to Western blot (WB) using different primary and secondary antibodies. WB procedure was similar to that described elsewhere [23]. Quantification of WB signals was done by image J (version 1.53) as described previously [24]. Statistical analyses were completed by GraphPad Prism (version 9) software and data are shown as mean ± standard error of the mean (SEM) [5].

### 2.4. Antibodies

The following antibodies were used in this study: rabbit polyclonal anti-MeCP2 antibody (abn1728, 1:1000); rabbit polyclonal anti-GAPDH antibody (G9545, 1:5000); rabbit polyclonal anti-SNAP25 antibody (S9684, 1:1000) [Sigma-Aldrich, St. Louis, MO, USA]; rabbit monoclonal anti-BDNF antibody (ab108319, 1:1000); rabbit polyclonal anti-MBP antibody (ab40390, 1:1000) [Abcam, Cambridge, UK]; rabbit IgG anti-S6 Ribosomal Protein antibody (5G10, 1:1000); rabbit anti-phospho-S6 Ribosomal Protein antibody (p-RSP6)(Ser235/236) (2211, 1:1000); rabbit anti-SQSTM1/P62 antibody (5114S, 1:1000); rabbit IgG anti-LC3A/B antibody (12741P, 1:1000) [Cell Signaling, Danvers, MA, USA]; mouse monoclonal anti-PSD95 antibody (MA1045, 1:1000) [Invitrogen, Waltham, MA, USA]; secondary HRP-linked antibodies (Anti-Rabbit IgG, 7074P2 and Anti-Mouse IgG, 7076P2) [Cell Signaling].

## 3. Results

### 3.1. Metformin Treatment Leads to Sex-Dependent Change in Phosphorylated RPS6 in the Liver, without Affecting the Level of Unphosphorylated RPS6

Our previous studies in human RTT brains showed increased phosphorylation of the two complexes of the mammalian target of rapamycin (mTOR); mTORC1 and mTORC2 (S2448 and S2481) [23]. Metformin is an anti-diabetic drug that is reported to be an mTOR inhibitor [25,26]. Phosphorylation of the ribosomal protein S6 has been generally accepted and commonly measured as the readout of the mTORC activity [27]. Thus, we first aimed to study the role of metformin in inducing RPS6 phosphorylation.

We randomly assigned male and female C57BL/6 mice to vehicle and metformin treatment groups, with six mice in each group (total of 24 mice). The mice were subjected to IP injection with metformin (200 mg/kg) or vehicle for 21 days, with daily treatments (Figure 1A). At the end of the treatments, mice were euthanized, and liver tissues were dissected along with specific brain regions (discussed later). Regarding body weight, daily monitoring showed no significant difference between vehicle- and metformin-treated mice in either the male or female group. However, a sex-dependent difference between male and female mice of the same age was observed, with males being heavier than females (**** *p* < 0.0001). Additionally, the daily average body weight of males was significantly higher than that of females (** *p* < 0.01, *** *p* < 0.001, **** *p* < 0.0001) (Figure 1B).

**Figure 2:**
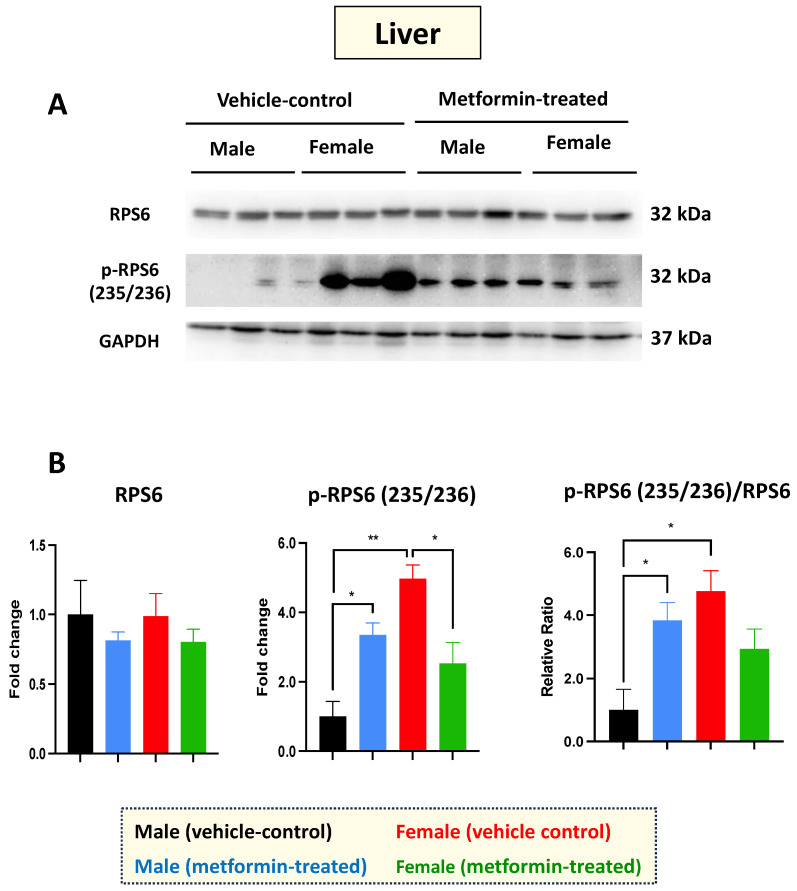
Metformin-induced sex-dependent changes in the level of phosphorylated ribosomal protein S6 in the murine liver. (**A**) Western blot detection of the indicated proteins showing the effect of metformin on the murine hepatic S6 and p-S6 (235/236). (**B**) Quantification of data by normalization of relative band intensities with their corresponding GAPDH signals. Fold change calculated relative to average fold change of male vehicle control. Sex-differences analysis indicated a significantly higher level of phosphorylated p-RPS6 (235/236) (** *p* < 0.01) in female mice compared with males. Metformin significantly induced (* *p* < 0.05) the level of phosphorylated p-RPS6 (235/236) in males while inhibiting (* *p* < 0.05) p-RPS6 in females. Additionally, metformin significantly induced (* *p* < 0.05) the relative ratio of p-RPS6 (235/236)/S6 in males. The analysis of data was completed with unpaired *t*-test and is presented by mean ± SEM with N = 3. The levels of significance are shown by * *p* < 0.05 and ** *p* < 0.01. Original images can be found in Figure S1.

Western blot experiments with total cell extracts from the liver tissues were performed with antibodies against the non-phosphorylated and phosphorylated RPS6 at the amino acids 235/236. Regarding RPS6 basal levels, we did not detect any difference between male and female mice in either the vehicle or the metformin-treated group. However, a significant increase in p-RPS6 (235/236) was detected in the livers of male mice (* *p* < 0.05), with the opposite impact observed in the female group (* *p* < 0.05), where we detected a significant decrease in p-RPS6 (235/236) in the metformin-treated female mice compared to those that received vehicle injection. In addition, we observed a significant sex difference regarding the basal levels of p-RPS6 (235/236), with higher levels in the livers of female mice compared to males (** *p* < 0.01) (Figure 2A,B). Measurements of the phosphorylated RPS6 (p-RPS6) (235/236) to unphosphorylated RPS6 ratio followed a similar pattern, increasing significantly in male mice and showing a significant difference between the two sexes (* *p* < 0.05). While a reduced phosphorylated RPS6 (235/236) to unphosphorylated RPS6 ratio was also detected in the livers of metformin-treated female mice, this difference did not reach statistical significance. Taken together, our data suggest that metformin has a molecular impact on RPS6 and its phosphorylated form in a sex-dependent manner, without impacting weight.

### 3.2. Metformin Induces MeCP2 in the Hippocampus of Male Mice, While Inhibiting pre-proBDNF and proBDNF in Female Mice

Rett Syndrome is associated with aberrant molecular and functional properties of the hippocampus, a particular brain region that controls memory formation [21,28]. In this regard, the *MECP2-BDNF* homeostatic regulation is impaired in RTT brain tissues [8]. Of note, increased MeCP2 and/or BNDF have been highlighted to have therapeutic value in the context of Rett Syndrome [2,29]. Thus, we aimed to study the level of MeCP2 and BDNF in the metformin-treated hippocampus tissues of male and female mice compared to male and female control (vehicle) mice group.

**Figure 3:**
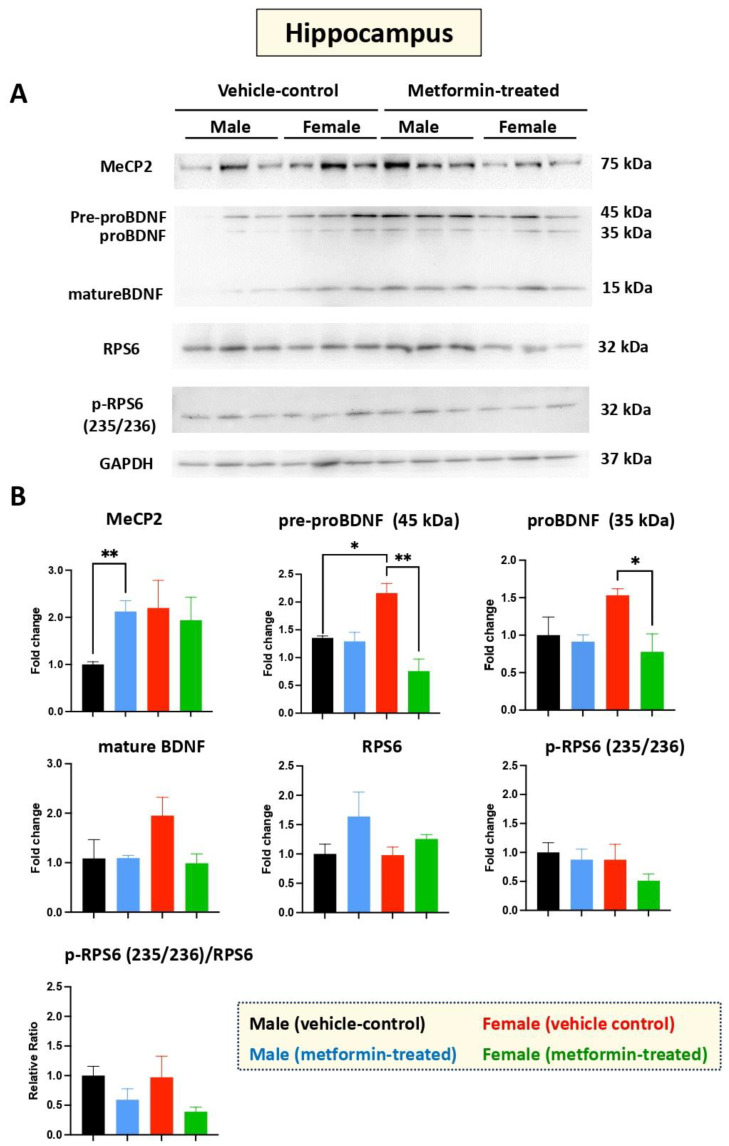
Metformin-mediated induction of MeCP2 in the male murine hippocampus, and inhibition of pre-proBDNF and proBDNF in the female murine hippocampus. (**A**) Representative immunoblots of the effect of metformin on murine hippocampal proteins including MeCP2, pre-proBDNF, proBDNF, mature BDNF, RPS6, and p-RPS6 (235/2536). (**B**) Quantification of data by normalization of relative band intensities with their corresponding GAPDH signals. Fold change calculated relative to average fold change of male vehicle control. Metformin significantly induced MeCP2 (** *p* < 0.01) in the hippocampus of male mice, but not in female mice. Further, metformin significantly reduced expression of pre-proBDNF (45 KDa) and proBDNF (35 KDa) in female mice. Sex-dependent differences indicated a higher (* *p* < 0.05) level of pre-proBDNF (45 kDa) in females compared with males. Data analysis was completed with unpaired *t*-test and is presented by mean ± SEM with N = 3. Also, the levels of significance are shown by * *p* < 0.05 and ** *p* < 0.01. Original images can be found in Figure S2.

Western blot experiments with an antibody against MeCP2 protein showed that levels are significantly (** *p* < 0.01) induced by metformin in the hippocampus of male mice. However, we did not observe any significant difference in MeCP2 levels between the metformin-treated female mice compared with the vehicle-treated female mice (Figure 3A,B).

Additional experiments were conducted to study the effect of metformin versus vehicle treatment on BDNF and its precursors, pre-proBDNF and proBDNF. No change in BDNF protein levels was observed in the hippocampus for male mice treated with metformin or vehicle. However, reduced levels of pre-proBDNF (** *p* < 0.01) and proBDNF (* *p* < 0.05) in the hippocampus were observed for female mice treated with metformin. Additionally, a trend of reduction in mature BDNF was noted in the metformin-treated female mice, though it was not of statistical significance. We also noted a significantly higher level of pre-proBDNF in the hippocampus of female versus male mice (* *p* < 0.05), which may not be biologically relevant, as the levels of proBDNF and mature BDNF were not statistically significant, although a similar higher trend in females was observed. Measurement of the levels of RPS6, in both unphosphorylated and phosphorylated form (235/236), did not show any significant difference between the metformin-treated mice and the control-vehicle groups (Figure 3A,B).

Taken together, our results show that metformin induces MeCP2 in the hippocampus of male mice, but not in female mice. We also show that metformin reduces the level of pre-proBDNF and proBDNF in the hippocampus of female mice, but this effect is not significantly detected in the levels of mature BDNF, suggesting additional regulatory levels.

### 3.3. The Impact of Metformin on the MeCP2, BDNF, and RPS6 in the Cerebellum and Thalamus

**Figure 4:**
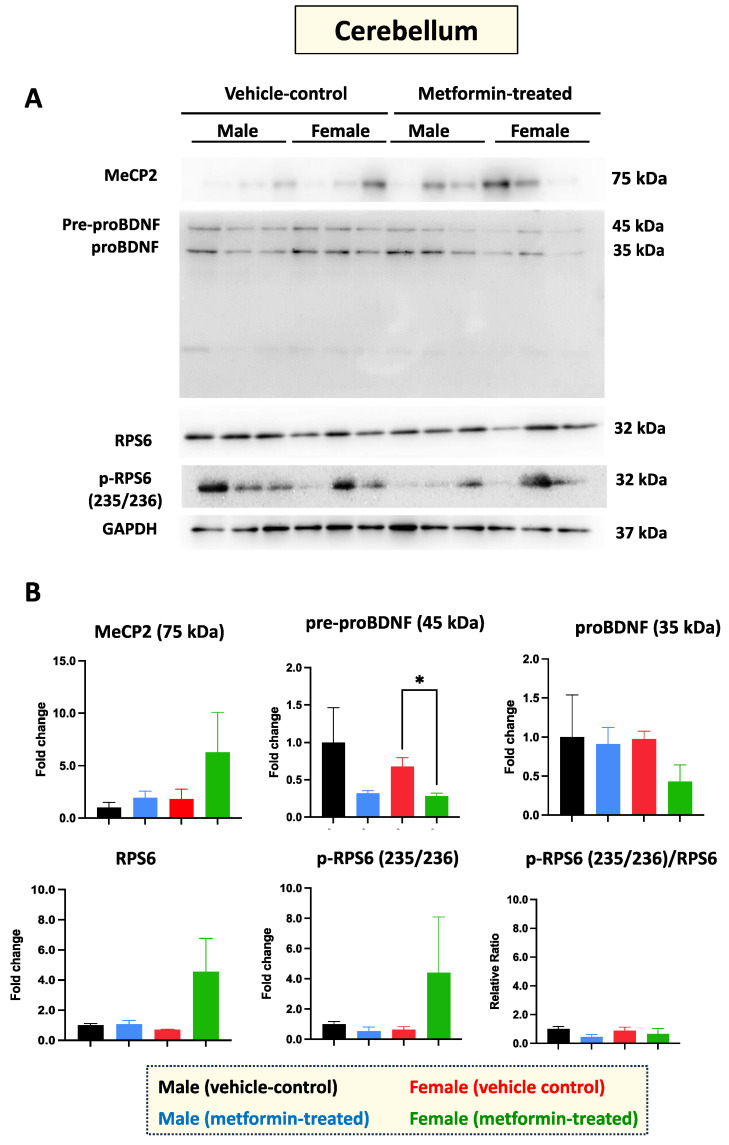
Metformin-mediated reduction of pre-proBDF (45 KDa) expression in female murine cerebellum. (**A**) Western blot experiments showing the effect of metformin on murine cerebellum proteins including MeCP2, pre-proBDNF, proBDNF, mature BDNF, RPS6, and p-RPS6 (235/2536). (**B**) Quantification of data by normalization of relative band intensities with their corresponding GAPDH signals. Fold change calculated relative to average fold change of male vehicle control. Significant reduction in pre-proBDNF (* *p* < 0.05) was detected in the cerebellum of female mice, but not in that of male mice. Data analysis was completed with unpaired *t*-test and presented by mean ± SEM with N = 3. The level of significance is shown by * *p* < 0.05. Original images can be found in Figure S3.

RTT patients show impaired molecular properties of neurons in the cerebellum [23]. Therefore, we aimed to study the molecular impact of metformin in the cerebellum of male and female mice. Total protein extracts from the cerebellum tissue of three vehicle-control male and three vehicle-control female mice were compared to those from the cerebellum tissue of metformin-treated mice. Western blot analysis with antibodies against MeCP2, BDNF, RPS6, and phosphorylated RPS6 (235/236) did not show any significant difference between control and metformin-treated tissues, except that pre-proBDNF was decreased by metformin in females (* *p* < 0.05) (Figure 4A,B). However, a trend of increased MeCP2 levels by metformin was detected in the cerebellum of both the male and female mice that was not statistically significant. In the cerebellum tissue of RTT patients and non-RTT controls, the levels of mature BDNF are lower than in other brain regions [8]. In our studies, the level of mature BDNF in the cerebellum was found to be below the detection level of Western blotting. The levels of both phosphorylated and unphosphorylated RPS6 in the cerebellum tissues revealed no significant difference between the metformin-treated mice and the control groups, with non-significant variation between different groups in both male and female mice (Figure 4A,B).

**Figure 5:**
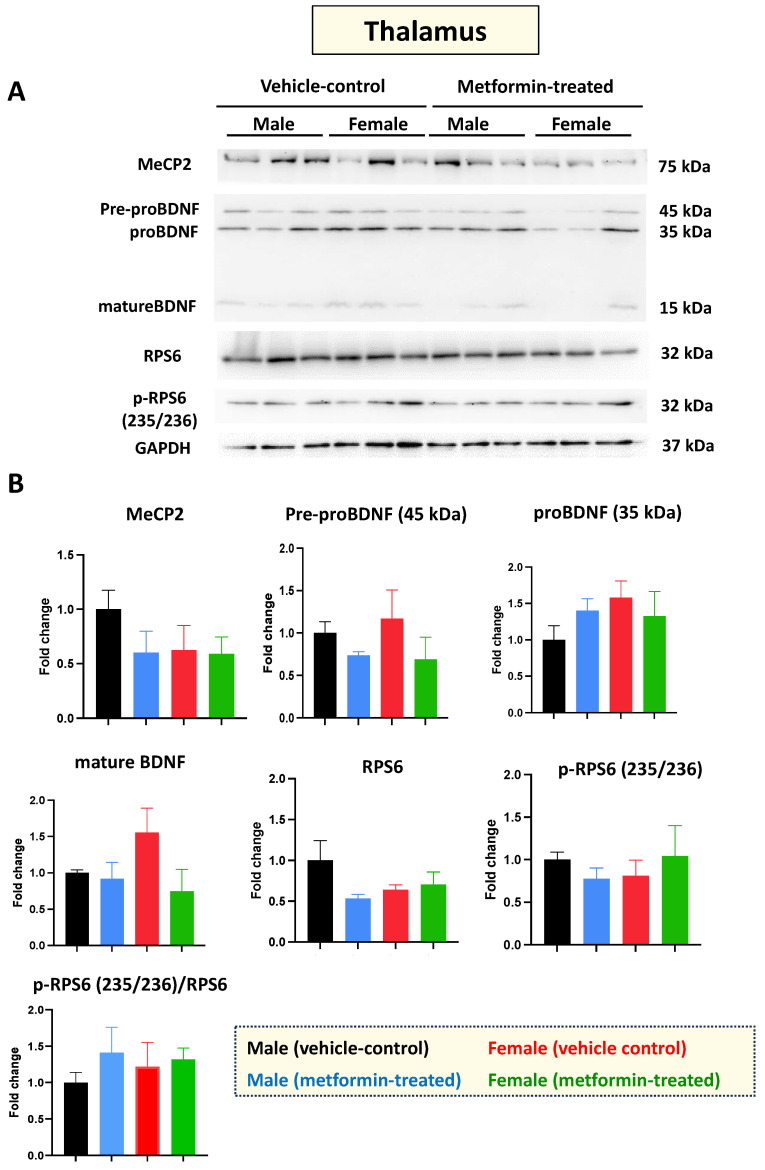
Metformin treatment did not change the level of the studied proteins in the thalamus. (**A**) Western blot detection in the thalamus of the male and female mice for MeCP2, pre-proBDNF, proBDNF, mature BDNF, RPS6, and p-RPS6 (235/2536). (**B**) Quantification of data by normalization of relative band intensities with their corresponding GAPDH signals. Fold change calculated relative to average fold change of the male vehicle control. Data analysis was done with unpaired *t*-test and presented by mean ± SEM with N = 3. Original images can be found in Figure S4.

*Mecp2*-deficient mice show impaired molecular and functional properties in the thalamus [30]. So, next, we investigated the molecular impact of metformin in the thalamus, for both male and female mice. As with the cerebellum, Western blot analysis of protein extracts from thalamus tissues with antibodies against MeCP2, BDNF, RPS6, and phosphorylated RPS6 (235/236) did not show any significant difference between the control and metformin-treated groups (Figure 5A,B). Collectively, we did not observe any significant impact of metformin on the level of these studied proteins in the cerebellum or thalamus in either sex of mice.

### 3.4. The Molecular Impact of Metformin in the Murine Frontal Cortex

**Figure 6:**
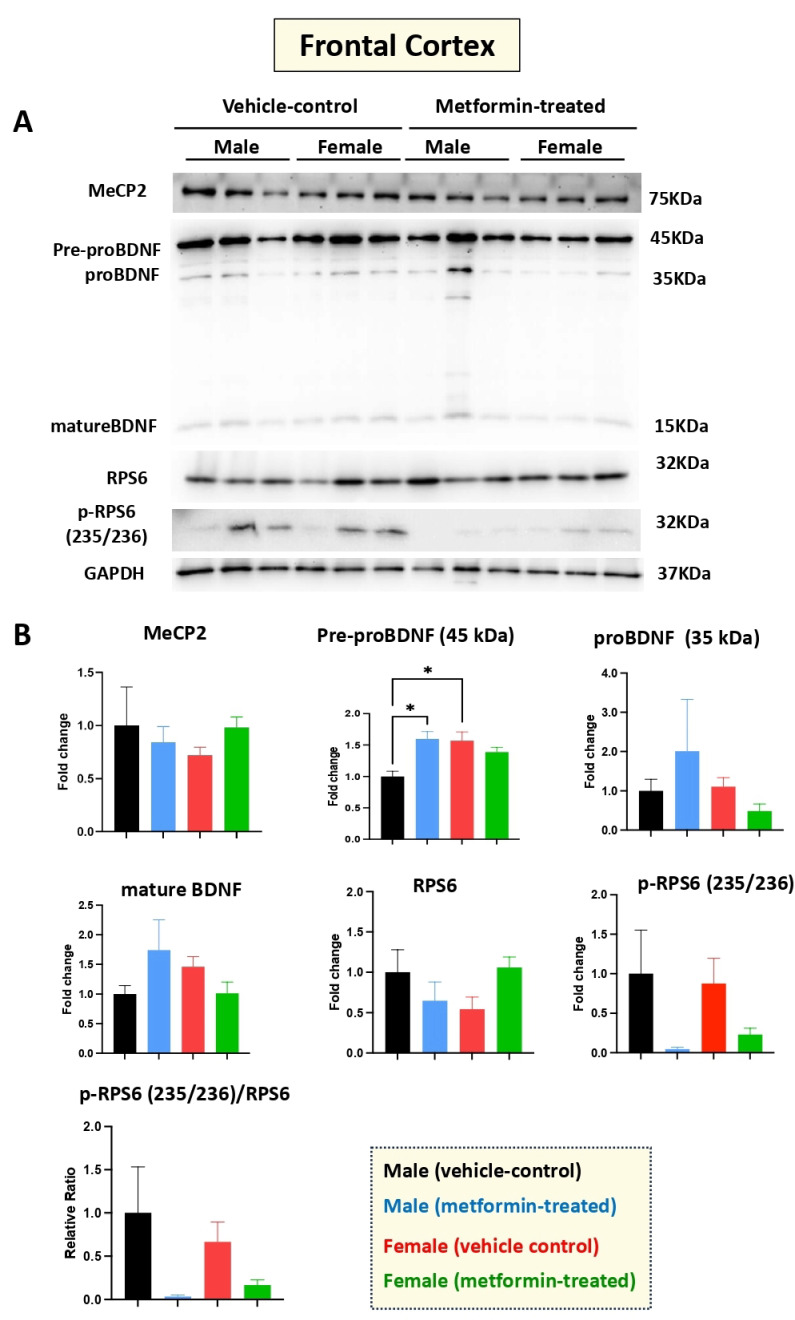
Metformin treatment did not change the level of proteins of the murine frontal cortex, except for pre-proBDNF. (**A**) Western blot detection of the effect of metformin on the indicated proteins in the frontal cortex for male and female mice. (**B**) Quantification of data by normalization of relative band intensities with their corresponding GAPDH signals. Fold change calculated relative to average fold change of male vehicle control. The level of pre-proBDNF in female vehicle is higher compared with male vehicle. Metformin significantly induced the level of male pre-proBDNF, while it did not affect the level of other proteins; however, slight reduction in the level of p-RPS6 (235/236) and the ratio of p-RPS6/RPS6 were observed in metformin-treated mice. Data analysis was done by unpaired *t*-test and presented by mean ± SEM with N = 3. The level of significance is shown by * *p* < 0.05. Original images can be found in Figure S5.

Human RTT patients show gross volumetric changes in the frontal cortex [31] along with grey-white-matter molecular abnormalities [32]. Thus, we aimed to study the molecular impact of metformin in the frontal cortex in the male and female mice groups. Our Western blot experiments with protein extracts from the tissues of metformin-treated mice versus controls did not show any significant difference regarding the levels of MeCP2, BDNF (proBDNF, and mature BDNF), RPS6, and p-RPS6 (235/236) (Figure 6). However, a significantly higher level of pre-proBDNF was observed in the frontal cortex of the female vehicle controls compared to the male vehicle controls. Further, a similar trend of reduced levels of p-RPS6 (235/236) was detected in the collected frontal cortex for both male and female mice that were treated with metformin, compared to controls. This variation was similar in both sexes but did not reach statistical significance in either sex.

**Figure 7:**
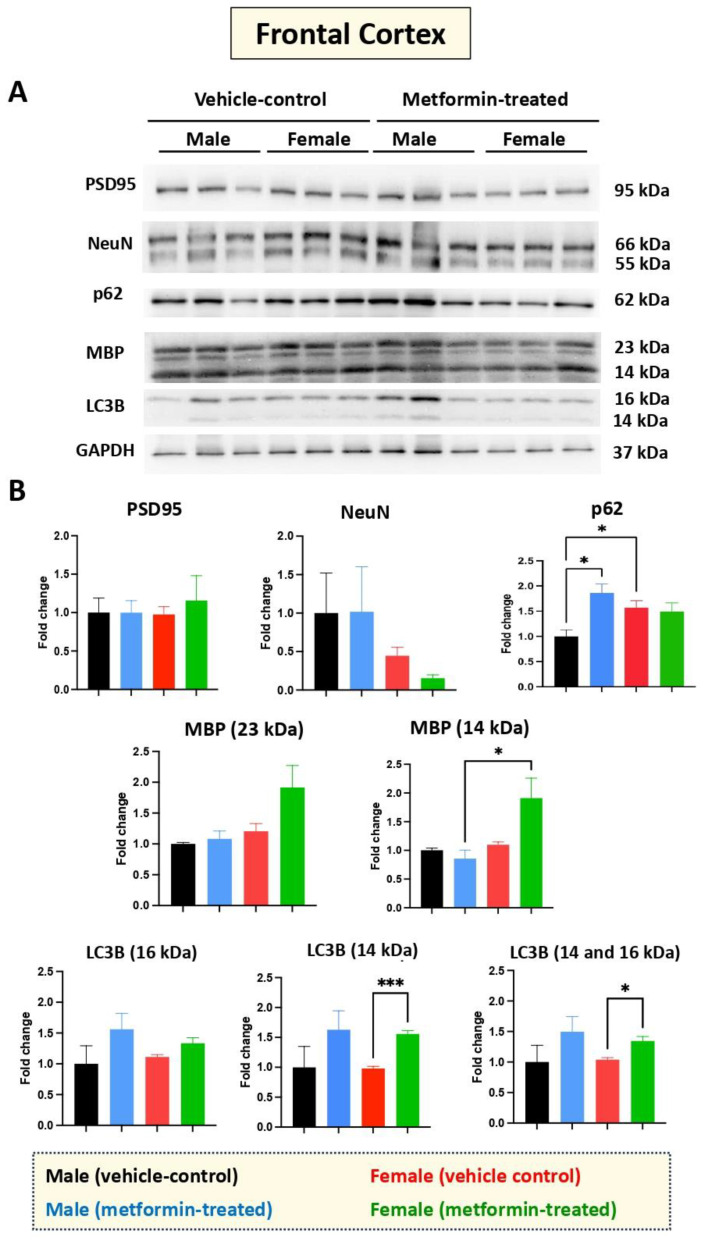
Metformin-mediated effects on cell type-specific and autophagy markers in the murine frontal cortex. (**A**) Western blot detection of indicated proteins for the effect of metformin on the murine frontal cortex for male and female mice. (**B**) Quantification of data by normalization of relative band intensities with their corresponding GAPDH signals. Fold change calculated relative to average fold change of male vehicle control. The level of P62 in female vehicle is higher compared to male vehicle; however, metformin-mediated induction of P62 was detected in male mice, but not in females. Metformin significantly induced MBP (14 kDa) (* *p* < 0.05) in the frontal cortex for the metformin-treated female mice as compared to male mice. Additionally, significant induction of LC3B (14 kDa) (*** *p* < 0.001) was observed in the metformin-treated female mice compared to female vehicle-control mice. Data analysis data was done with unpaired *t*-test and presented by mean ± SEM with N = 3. The level of significance is shown by * *p* < 0.05. Original images can be found in Figure S6.

**Figure 8:**
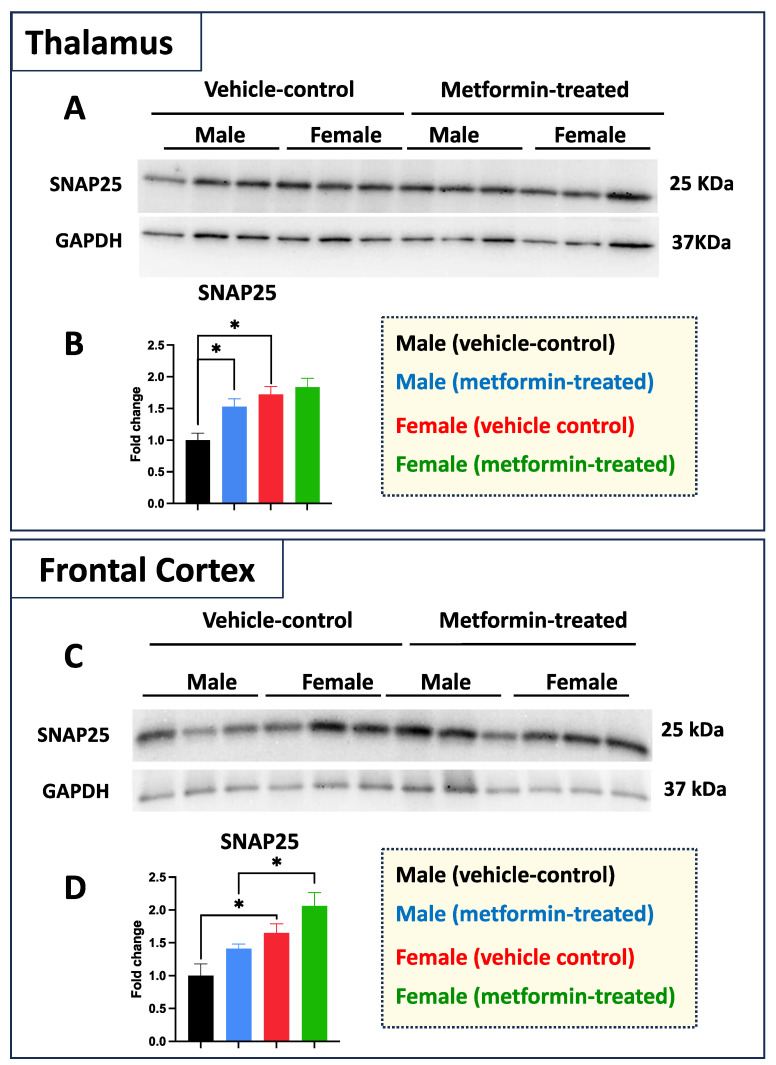
Metformin effect on pre-synaptic proteins is sex- and brain region-dependent in murine thalamus and frontal cortex. (**A**) Western blot analysis of the effect of metformin on SNAP25 protein levels in the murine thalamus. (**B**) Quantification of data by normalization of relative band intensities with their corresponding GAPDH signals. Fold change calculated relative to average fold change of male vehicle control. A significantly higher level of thalamic SNAP25 was detected in females compared to males. In addition, a metformin-mediated induction (* *p* < 0.05) of SNAP25 was detected in the male thalamus tissues as compared to male vehicle control. (**C**) Western blot analysis of the effect of metformin on SNAP25 protein level in the murine frontal cortex. (**D**) Quantification of data by normalization of relative band intensities with their corresponding GAPDH signals. Fold change calculated relative to average fold change of male vehicle control. Sex-differences analysis showed a higher level of SNAP25 protein in female mice (* *p* < 0.05) as compared to their male counterparts. Additionally, a metformin-mediated induction (* *p* < 0.05) of SNAP25 protein levels was detected in murine cortical tissues of females as compared to males. Data analysis was completed with unpaired *t*-test and presented by mean ± SEM with N = 3. Also, the levels of significance are shown by * *p* < 0.05. Original images can be found in Figure S7.

While we did not see any impact of metformin in most of the aforementioned proteins, except for pre-proBDNF, we decided to study the levels of selected additional proteins. Western blot analysis was performed for PSD95 (post-synaptic protein), NeuN (neuronal-specific marker), MBP (glial marker for oligodendrocytes), as well as P62 and LC3B (autophagy factors) (Figure 7). No significant change was detected for levels of PSD95, NeuN, and MBP in metformin-treated samples compared to vehicle control tissues. However, a trend of increased LC3B level was detected in the tissue of both male and female metformin-treated mice compared to controls; that reached to statistical significance in female tissues at 14 kDa. As neuronal circuits may depend on both the pre-synaptic and post-synaptic proteins, we studied the impact of metformin treatment on the pre-synaptic protein SNAP25. Analyzing frontal cortex tissues showed an increase trend of SNAP25 in both male and female mice that did not reach to statistical significance. We observed a statistically significant higher SNAP25 levels in the frontal cortex for the females compared to the males in vehicle-treated and metformin-treated mice (* *p* < 0.05). In addition to the frontal cortex, we randomly examined another brain region (thalamus) to study if there is any differential basal level of SNAP25. Our results indicated that, as with the frontal cortex, female tissues have a higher level of SNAP25 compared to male tissues (* *p* < 0.05). These results show that there might be some differential basal level of pre-synaptic proteins in between male and females at least in these two selected murine brain regions (Figure 8).

## 4. Discussion

Although there is currently no cure for RTT, several therapeutic avenues have been explored—the most recent being the Food and Drug Administration (FDA)-approved drug, trofinetide [33]. Despite its therapeutic potential, mild-to-severe adverse side effects have been reported in relation to the use of trofinetide for RTT patients, including diarrhea, seizures, and vomiting [33]. Thus, there remains a need for RTT treatment options that have low-to-no adverse side-effects for the affected patients. Considering the neurometabolic component of RTT, particularly the impaired glucose metabolism in the brains of RTT patients, metformin (1,1-dimethyl biguanide hydrochloride) has emerged as a potential therapeutic candidate for RTT [10,34,35]. Metformin is the first-line therapy option for the treatment and management of type 2 diabetes [36]. In recent years, however, metformin has been repurposed for the treatment of other human ailments such as cardiovascular, renal, and liver diseases as well as several types of cancers [37]. Studies suggest that metformin, which easily penetrates the BBB, may offer therapeutic effect in the context of neurological disorders, including Fragile X syndrome, Alzheimer’s disease, Parkinson’s disease, Huntington’s disease, and autism spectrum disorders [16,38,39,40,41]. While several lines of evidence have indicated a rescue in oxidative stress, aberrant mitochondrial activity, and impaired cognitive function as well as improved neurogenesis and spatial memory formation in mouse models of RTT, the molecular impact of metformin treatment in the context of RTT required further exploration [11,42,43]. The present study provides insight into the sex- and brain-region-dependent molecular impact of metformin in mice.

One generally accepted mechanistic action of metformin in the liver involves inhibition of hepatic mTORC1 signaling with subsequent phosphorylation of the mTORC1 downstream target RPS6 [44]. Thus, to determine the action of metformin in the liver, we compared the protein expression levels of phosphorylated RPS6 in both male and female mice. In our studies, metformin induced phosphorylation of RPS6 at amino acid residues 235/236 in a sex-dependent manner. Specifically, metformin induced RPS6 phosphorylation in the liver of male mice while this phosphorylation was reduced in female mice. As the functional role of RPS6 relates to ribosome biogenesis, phosphorylation of this protein may be an indication of inhibited protein translation or protein translation initiation. Thus, the results suggest that metformin acts in a sex-dependent manner to potentially inhibit protein translation via the mTORC1 signaling cascade in the livers of male mice.

Sex-dependent differences were also observed in the hippocampus of male and female mice treated with metformin. In one instance, metformin induced MeCP2 hippocampal expression in male, but not female, mice. As the restoration of MeCP2 protein levels is a favorable therapeutic avenue in the treatment of RTT, the induction of MeCP2 levels by metformin may be applicable for neurological diseases with MeCP2 loss-of-function mutations such as RTT, or decreased protein expression of MeCP2. Further, our Western blot analyses indicate reductions in both pre-proBDNF and proBDNF in female mice. Typically, BDNF (mature BDNF) is initially synthesized as precursor pre-proBDNF and later becomes proBDNF following peptide cleavage. The reduced levels of both BDNF precursors may represent enhanced conversion to mature BDNF or reduced transcription of *Bdnf* following treatment with metformin. Interestingly, recent studies from our lab using human brain cells (*in vitro*) have shown that metformin transcriptionally and/or post-transcriptionally induces *BDNF* and *MECP2E1* transcripts [12]. Based on these findings, reductions in BDNF precursor(s) may lead to induced *Bdnf* and in turn, increased BDNF protein expression. However, we did not observe this theorized increase in BDNF in either sex of mice. Moreover, similar significant sex-dependent differences in the protein expression of phosphorylated RPS6, MeCP2, proBDNF and mature BDNF were not observed in the cerebellum or thalamus for either sex of mice. However, reductions in phosphorylated RPS6 were observed in the frontal cortex for both male and female mice.

Generally, trends of sex-dependent molecular profiles following treatment with metformin have been previously reported in the context of type 2 diabetes, neonatal stroke, and spinal cord injury [45,46,47]. As RTT primarily affects females, the sex-dependent findings of our study related to metformin treatment provides insight into a potential therapeutic strategy. Our sex-dependent findings are in line with sex-specific uses of metformin clinically, such as in the treatment of polycystic ovary syndrome and gestational diabetes in females [48,49]. Additionally, studies also suggest sex-dependent differences in metformin-associated drug reactions, hence the lower dosages of metformin that are prescribed to females [50]. Altogether, sex-dependent differences pertaining to the use and dosage of metformin in females indicate that metformin may be a candidate for the treatment of sex-linked diseases that primarily affect females, such as RTT. In the brain, metformin is widely recognized for its role in a plethora of processes such as BBB integrity, regulation of protein expression via autophagy, inhibition of reactive astrogliosis, and regulation of synaptic plasticity and transmission [13]. Coincidentally, many of these processes are reportedly impaired in RTT. 

## 5. Conclusions

Here, we studied the molecular impact of metformin at 200 mg/kg body weight in male and female mice. Our data supported a sex-specific impact in specific brain regions of the mice treated with metformin compared to control-vehicle-treated mice. In addition to MeCP2, BDNF, and RPS6 (phosphorylated and non-phosphorylated form), we also studied certain cell type-specific markers and autophagy proteins. Additionally, the pre-synaptic protein SNAP25 was significantly increased in the thalamus for the metformin-treated male mice compared to their male counterparts. Collectively, our results underscore the sex-dependent molecular impact of metformin in mice.

## Data Availability

All data related to this manuscript exists within this paper, and original image of Western blot experiments are provided as Supplementary Figures.

## Supplementary Materials

The following supporting information can be downloaded at: https://www.mdpi.com/article/10.3390/biom14040505/s1**.** Figure S1. Uncropped images of Western blot membranes for Figure 2. Figure S2. Uncropped images of Western blot membranes for Figure 3. Figure S3. Uncropped images of Western blot membranes for Figure 4. Figure S4. Uncropped images of Western blot membranes for Figure 5. Figure S5. Uncropped images of Western blot membranes for Figure 6. Figure S6. Uncropped images of Western blot membranes for Figure 7. Figure S7. Uncropped images of Western blot membranes for Figure 8.
