# Supplementary material for: Metformin Induces MeCP2 in the Hippocampus of Male Mice with Sex-Specific and Brain-Region-Dependent Molecular Impact
Source: Biomolecules. 2024 Apr 21;14(4):505. doi: 10.3390/biom14040505 (PMC11048179; doi:10.3390/biom14040505)
Supplement: Supplementary file 1 [file biomolecules-14-00505-s001.zip › biomolecules-2877013-supplementary.pdf]

Article

Metformin Induces MeCP2 in the Hippocampus of Male Mice with Sex-Specific and Brain-Region-Dependent Molecular Impact

Khatereh Saei Arezoumand, Chris-Tiann Roberts and Mojgan Rastegar *

Department of Biochemistry and Medical Genetics, Rady Faculty of Health Sciences, Max Rady College of Medi-cine, University of Manitoba, Winnipeg, MB R3E 0J9, Canada

* Correspondence: mojgan.rastegar@umanitoba.ca

Supplementary Figures

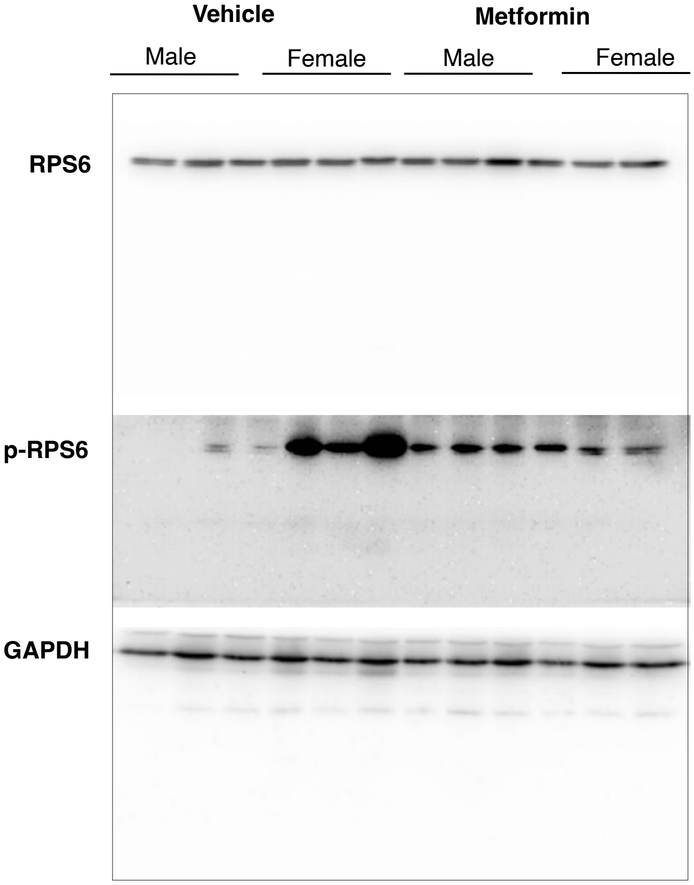

Supplementary Figure S1. Uncropped images of Western blot membranes for Figure 2. Note that all membranes were re-probed for GAPDH, but only one GAPDH representative is shown in Figure 2. Uncropped image of this GAPDH is shown in this supplementary Figure. All proteins were quantified using their respective GAPDH for the corresponding membranes. Signal quantifications are provided in Figure 2.

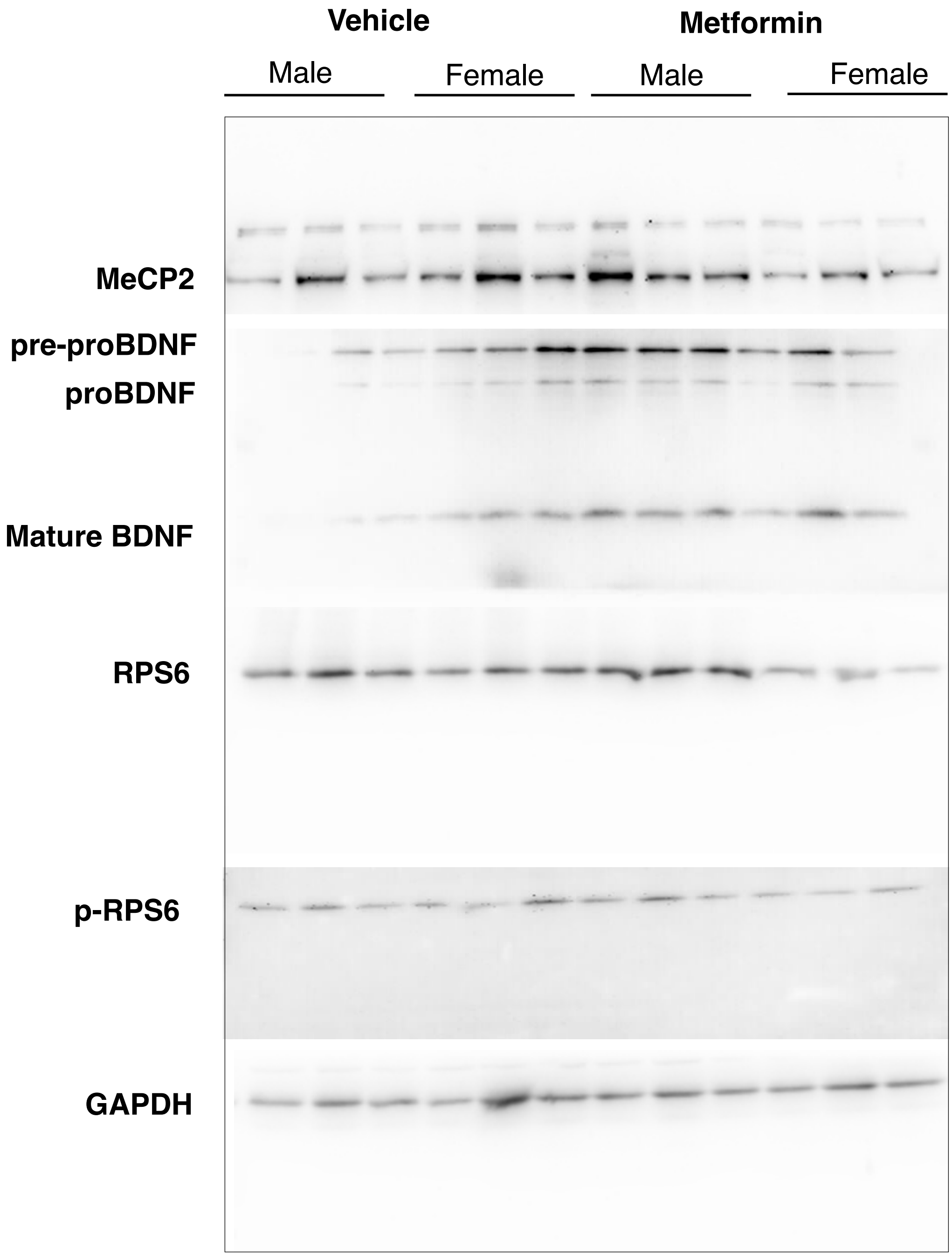

Supplementary Figure S2. Uncropped images of Western blot membranes for Figure 3. Note that all membranes were re-probed for GAPDH, but only one GAPDH representative is shown in Figure 3. Uncropped image of this GAPDH is shown in this supplementary Figure. All proteins were quantified using their respective GAPDH for the corresponding membranes. Signal quantifications are provided in Figure 3.

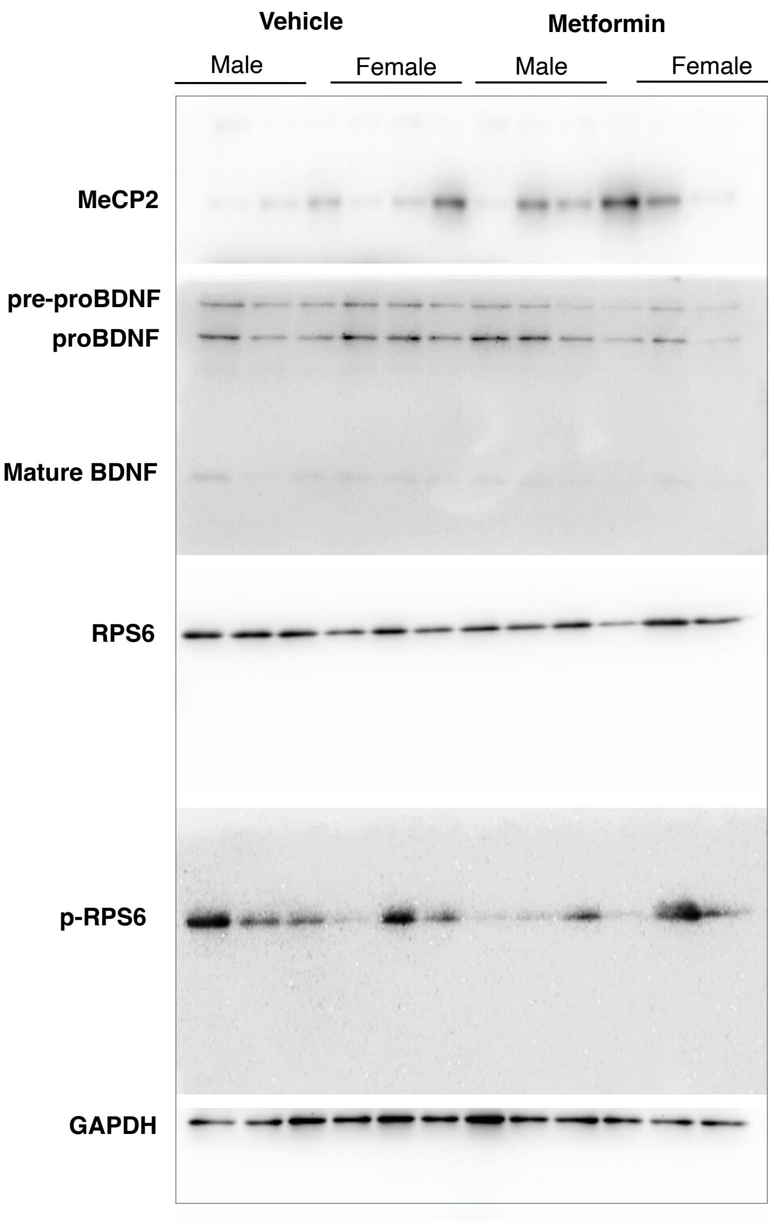

Supplementary Figure S3. Uncropped images of Western blot membranes for Figure 4. Note that all membranes were re-probed for GAPDH, but only one GAPDH representative is shown in Figure 4. Uncropped image of this GAPDH is shown in this supplementary Figure. All proteins were quantified using their respective GAPDH for the corresponding membranes. Signal quantifications are provided in Figure 4.

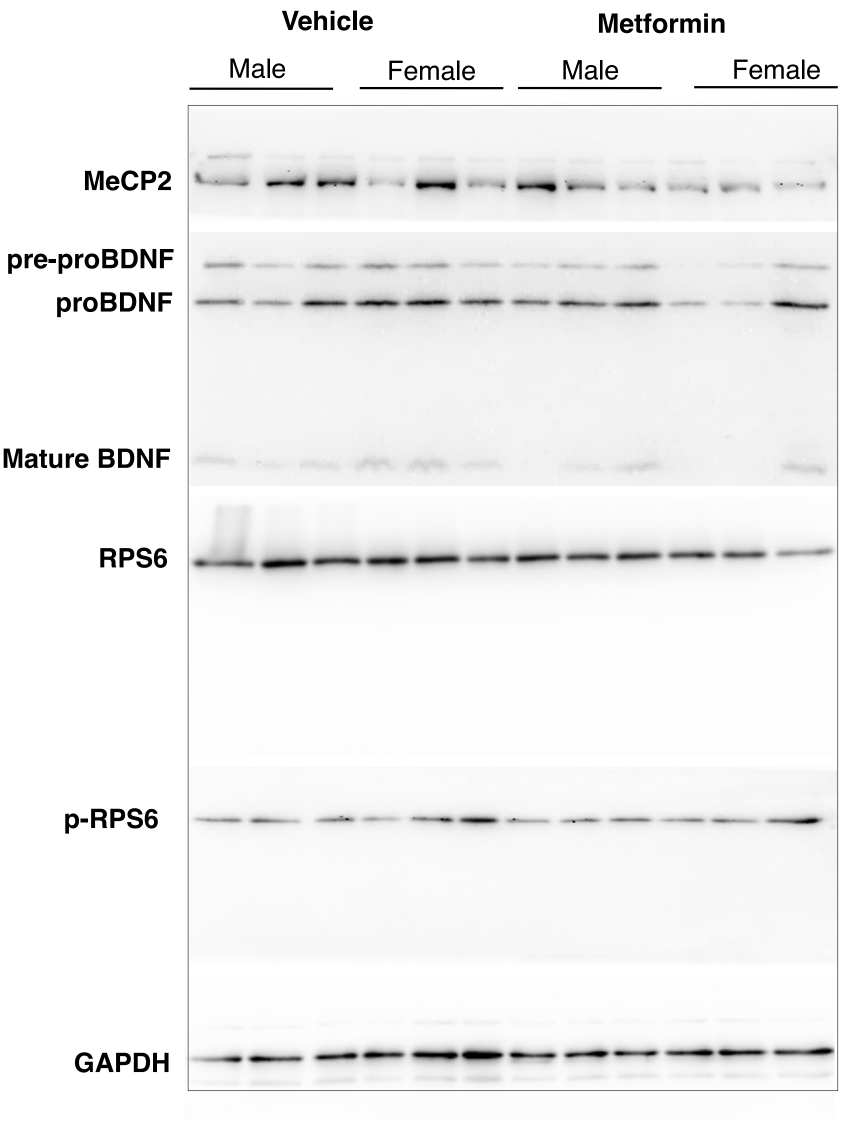

Supplementary Figure S4. Uncropped images of Western blot membranes for Figure 5. Note that all membranes were re-probed for GAPDH, but only one GAPDH representative is shown in Figure 5. Uncropped image of this GAPDH is shown in this supplementary Figure. All proteins were quantified using their respective GAPDH for the corresponding membranes. Signal quantifications are provided in Figure 5.

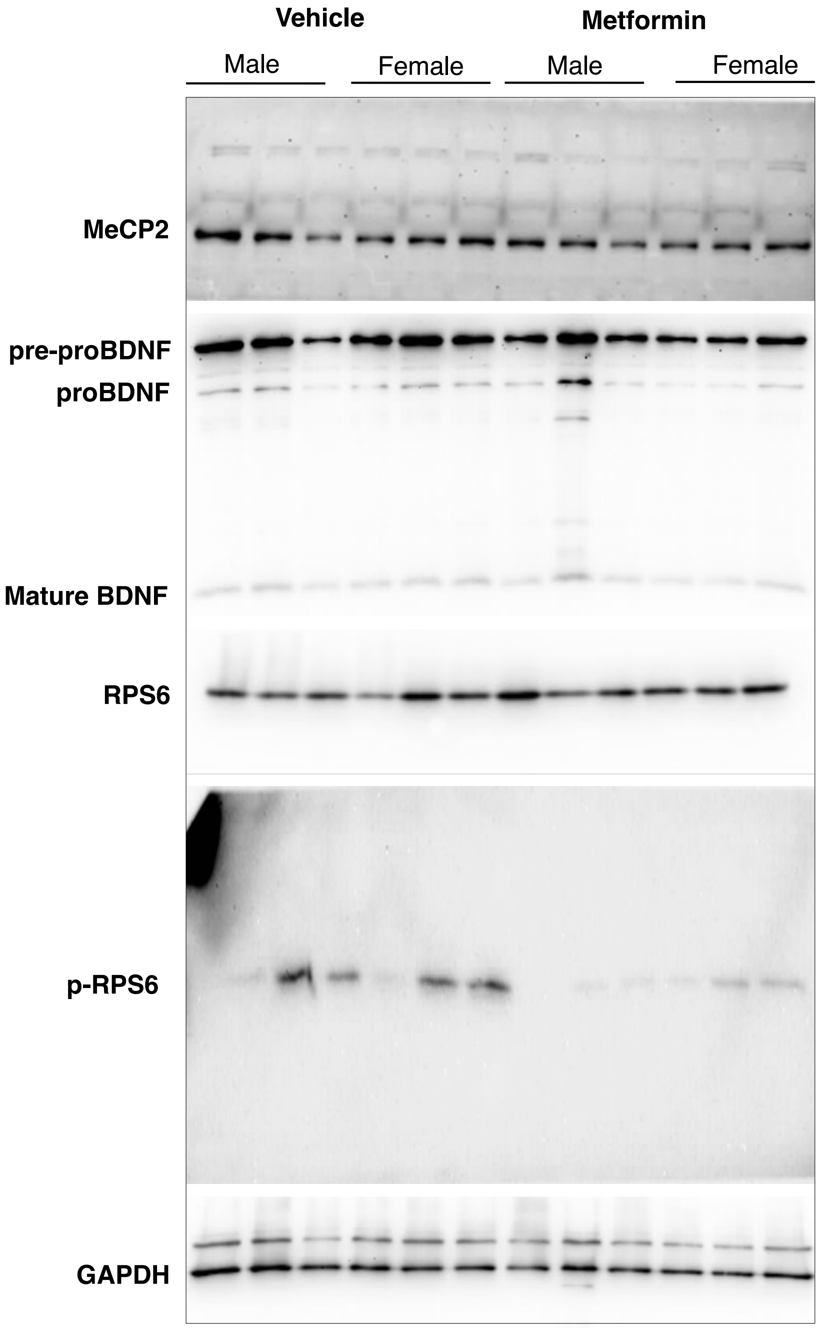

Supplementary Figure S5. Uncropped images of Western blot membranes for Figure 6. Note that all membranes were re-probed for GAPDH, but only one GAPDH representative is shown in Figure 6. Uncropped image of this GAPDH is shown in this supplementary Figure. All proteins were quantified using their respective GAPDH for the corresponding membranes. Signal quantifications are provided in Figure 6.

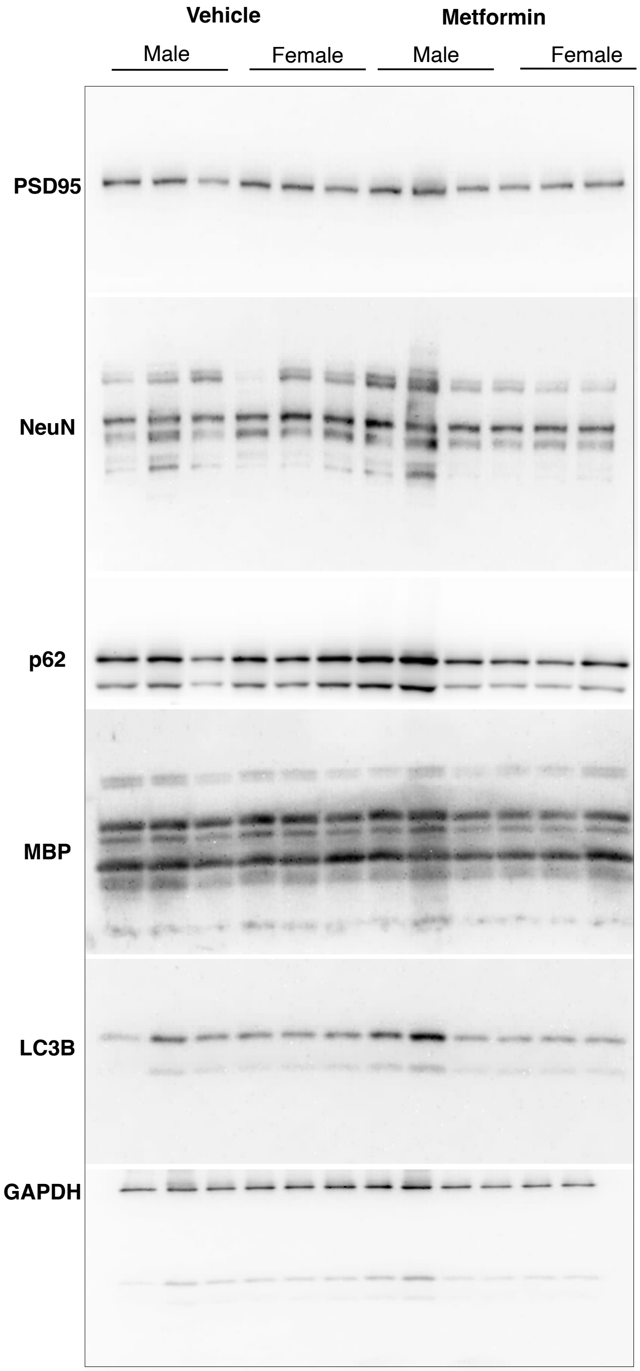

Supplementary Figure S6. Uncropped images of Western blot membranes for Figure 7. Note that all membranes were re-probed for GAPDH, but only one GAPDH representative is shown in Figure 7. Uncropped image of this GAPDH is shown in this supplementary Figure. All proteins were quantified using respective GAPDH for corresponding membranes. Quantifications are provided in Figure 7.

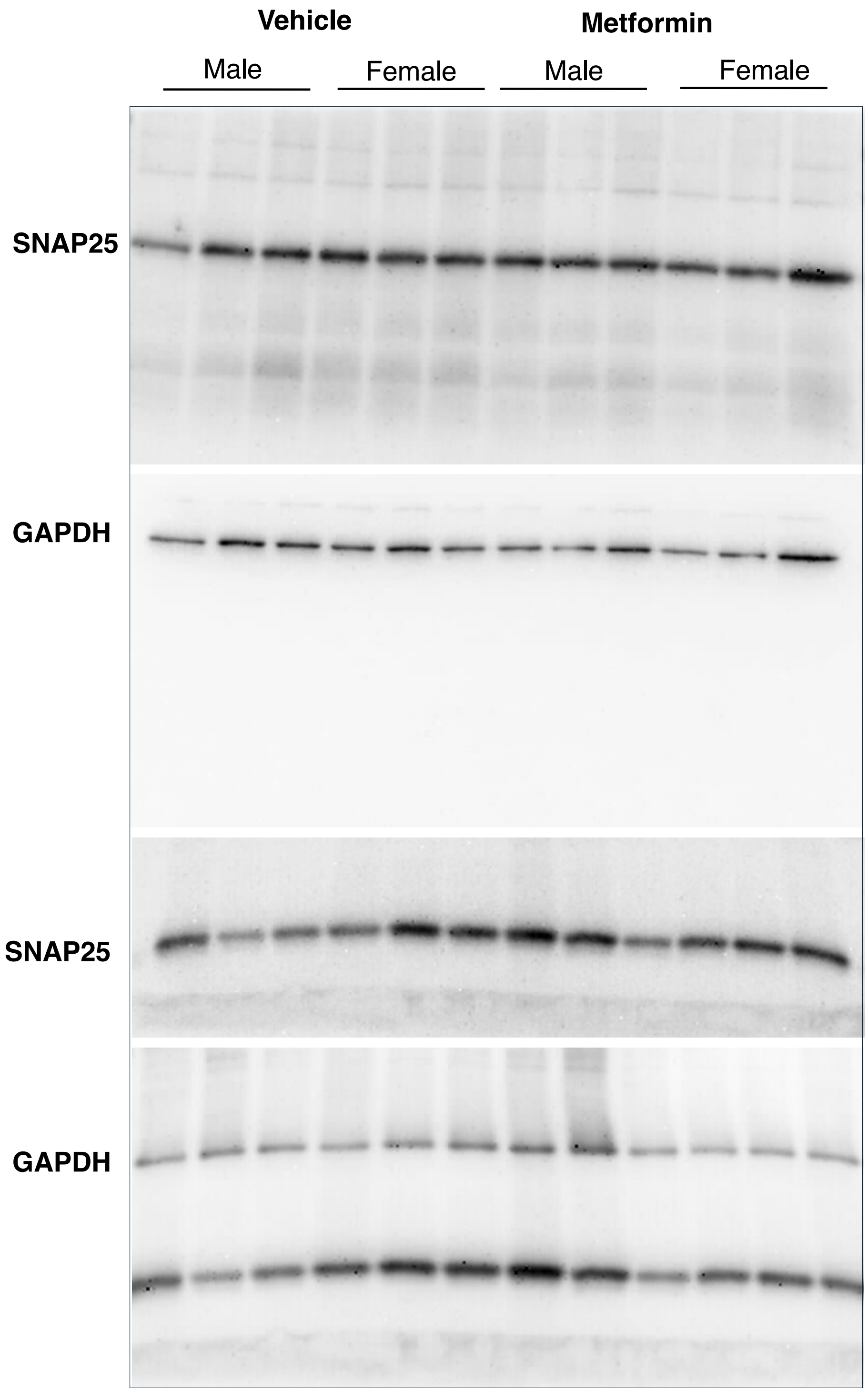

Supplementary Figure S7. Uncropped images of Western blot membranes for Figure 8. Uncropped image GAPDH corresponds to the SNAP25 membrane in each brain region (Thalamus on the top and Frontal Cortex in the bottom). Quantifications are provided in Figure 8.
